# The Non-Benzodiazepine Anxiolytic Drug Etifoxine Causes a Rapid, Receptor-Independent Stimulation of Neurosteroid Biosynthesis

**DOI:** 10.1371/journal.pone.0120473

**Published:** 2015-03-18

**Authors:** Jean Luc do Rego, David Vaudry, Hubert Vaudry

**Affiliations:** 1 Institute for Research and Innovation in Biomedicine (IRIB), University of Rouen, Mont-Saint-Aignan, France; 2 Regional Platform for Cell Imaging (PRIMACEN), International Associated Laboratory Samuel de Champlain, University of Rouen, Mont-Saint-Aignan, France; 3 Neurotrophic Factors and Neuronal Differentiation team, Inserm U982, University of Rouen, Mont-Saint-Aignan, France; University of Hong Kong, HONG KONG

## Abstract

Neurosteroids can modulate the activity of the GABA_A_ receptors, and thus affect anxiety-like behaviors. The non-benzodiazepine anxiolytic compound etifoxine has been shown to increase neurosteroid concentrations in brain tissue but the mode of action of etifoxine on neurosteroid formation has not yet been elucidated. In the present study, we have thus investigated the effect and the mechanism of action of etifoxine on neurosteroid biosynthesis using the frog hypothalamus as an experimental model. Exposure of frog hypothalamic explants to graded concentrations of etifoxine produced a dose-dependent increase in the biosynthesis of 17-hydroxypregnenolone, dehydroepiandrosterone, progesterone and tetrahydroprogesterone, associated with a decrease in the production of dihydroprogesterone. Time-course experiments revealed that a 15-min incubation of hypothalamic explants with etifoxine was sufficient to induce a robust increase in neurosteroid synthesis, suggesting that etifoxine activates steroidogenic enzymes at a post-translational level. Etifoxine-evoked neurosteroid biosynthesis was not affected by the central-type benzodiazepine (CBR) receptor antagonist flumazenil, the translocator protein (TSPO) antagonist PK11195 or the GABA_A_ receptor antagonist bicuculline. In addition, the stimulatory effects of etifoxine and the triakontatetraneuropeptide TTN, a TSPO agonist, were additive, indicating that these two compounds act through distinct mechanisms. Etifoxine also induced a rapid stimulation of neurosteroid biosynthesis from frog hypothalamus homogenates, a preparation in which membrane receptor signalling is disrupted. In conclusion, the present study demonstrates that etifoxine stimulates neurosteroid production through a membrane receptor-independent mechanism.

## Introduction

Etifoxine (2-ethylamino-6-chloro-4-methyl-4-phenyl-4H-3,1-benzoxazine hydrochloride; Stresam) is an anxiolytic and anticonvulsant drug of the benzoxazine family [1]. The anxiolytic-like properties of this non-benzodiazepine compound have been documented in both rodents [2,3] and humans [4–6]. In particular, etifoxine attenuates stress-induced anxiety-like behaviors [7,8]. Etifoxine is devoid of benzodiazepine-related side effects, such as sedation, amnesia, myorelaxation, tolerance and dependence [9–12] and thus etifoxine preserves psychomotor, attention and memory performances [4,6]. It has been recently shown that etifoxine displays potent regenerative and anti-inflammatory properties, and promotes functional recovery in experimental models of traumatic peripheral nerve injury [13,14]. Etifoxine also exerts anti-hyperalgesic effects in a preclinical model of toxic neuropathy [15].

Two main mechanisms may account for the anxiolytic action of etifoxine. On the one hand, etifoxine enhances GABAergic neurotransmission through allosteric interaction with the GABA_A_ receptor [3,16]. In fact, etifoxine preferentially activates GABA_A_ receptors that encompass the β2 and/or β3 subunits [17] that are not the target of benzodiazepines and neuroactive steroids. On the other hand, etifoxine activates the translocator protein 18 kDa (TSPO) [3,18], formerly termed peripheral-type benzodiazepine receptor (PBR) [19,20]. In support of this notion, etifoxine shows comparable efficacy to the benzodiazepine lorazepam in patients suffering from adjustment disorders with anxiety [6,21] and the TSPO antagonist PK11195 partly suppresses the effect of etifoxine on GABAergic transmission [3,18]. It has been proposed that the neurotrophic and neuroprotective effects of etifoxine could be mediated by TSPO, inasmuch as they are mimicked by selective ligands of TSPO, but not by GABA_A_ receptor agonists [13,14]. However, the molecular mechanism underlying the anxiolytic and neurotrophic effects of etifoxine remain poorly understood.

It is now firmly established that the central nervous system is able to synthesize biologically active steroids, called neurosteroids, that exert various behavioral activities [22–26]. In particular, the neurosteroids tétrahydroprogesterone (THP; also termed allopregnanolone), a 3α, 5α-reduced metabolite of progesterone (P), and dehydroepiandrosterone (DHEA) exert anxiolytic-like properties and thus mimic some of the effects of etifoxine [21,27–35]. Reciprocally, down-regulation of neuroactive steroid content in the plasma and cerebrospinal fluid are associated with emotional disorders, including depression and anxiety [36]. These observations suggest that neurosteroids could relay the anxiolytic effect of etifoxine. In support of this hypothesis, it has been shown that intraperitoneal administration of etifoxine in adrenalectomized and castrated rats results in a significant increase in brain concentrations of pregnenolone (Δ^5^P), P, dihydroprogesterone (DHP) and THP [18]. It has also been reported that the anxiolytic action of etifoxine is potentiated by THP suggesting that the two molecules may either bind on distinct sites on the GABA_A_ receptor, or act on different receptors [37,38]. Previous studies have shown that Δ^5^P and P [39–43], in very much the same as etifoxine [13,14], promote myelin repair after sciatic nerve injury. A concomitant increase in TSPO expression has been observed during regeneration of lesioned peripheral nerves [44–46] and neurons [19,47]. Indeed, it is now well established that TSPO plays a key role in the regulation of biosynthesis of neuroactive steroids in the central and peripheral nervous systems [48–52]. Collectively, these observations indicate that neurosteroids could be involved in some of the behavioral and neurochemical effects of etifoxine. However, little is known regarding the mechanisms through which etifoxine may regulate the production of neuroactive steroids in the central nervous system.

**Fig 1:**
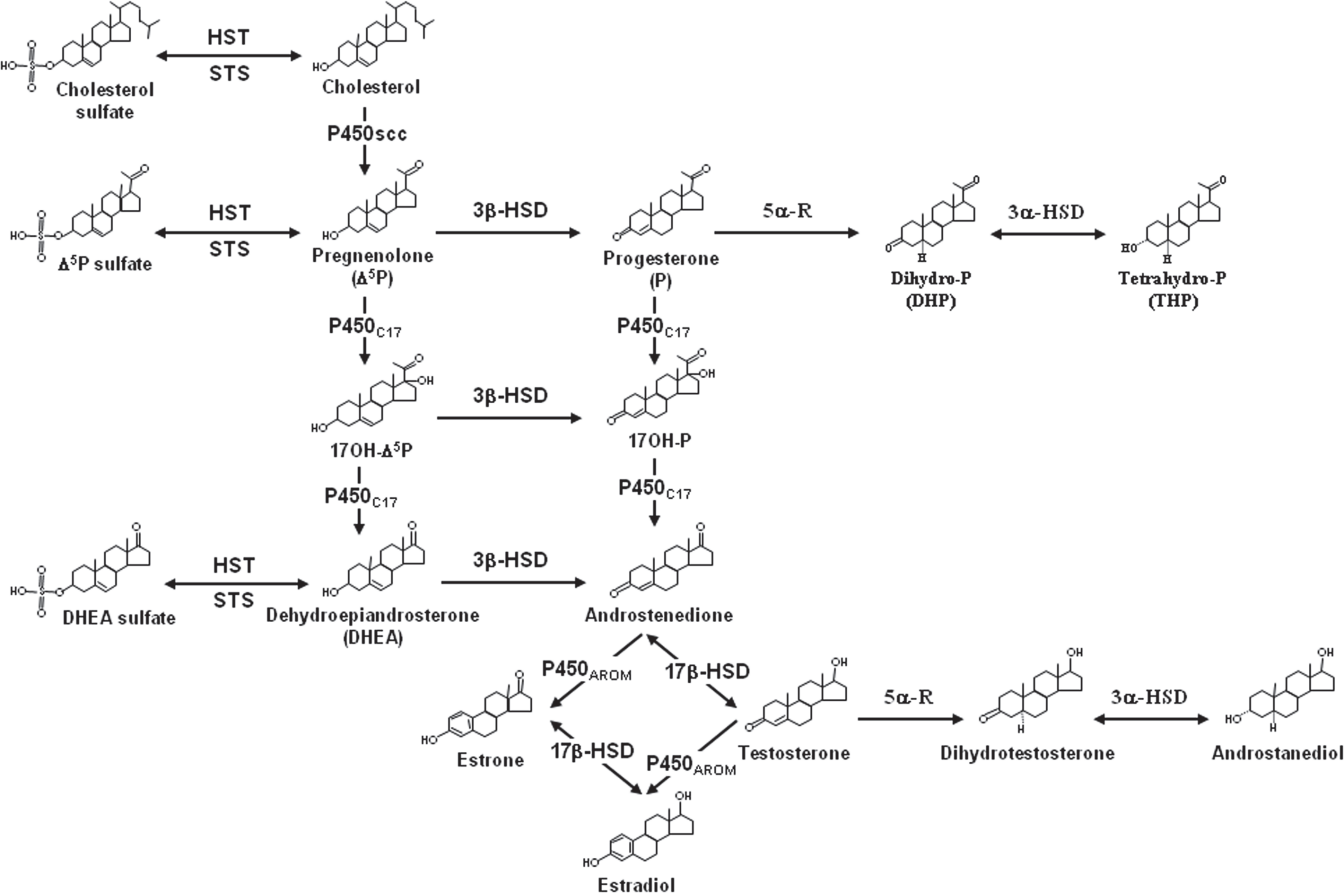
Simplified diagram recapitulating the biosynthetic pathways of neurosteroids in the brain of vertebrates. HST, hydroxysteroid sulfotransferase; P450_AROM_, cytochrome P450 aromatase; P450scc, cytochrome P450 side-chain cleavage; P450_C17_, cytochrome P450 17α-hydroxylase / C17,20-lyase; STS, sulfatase; 3α-HSD, 3α-hydroxysteroid dehydrogenase; 3β-HSD, 3β-hydroxysteroid dehydrogenase; 5α-R, 5α-reductase; 17β-HSD, 17β-hydroxysteroid dehydrogenase.

The frog brain, which expresses all major steroidogenic enzymes including cytochrome P450 side-chain cleavage (P450scc) [53], 3β-hydroxysteroid dehydrogenase / Δ^5^- Δ^4^ isomerase (3β-HSD) [54], cytochrome P450 17α-hydroxylase / C17, 20-lyase (P450_C17_) [55], 17β-hydroxysteroid dehydrogenase (17β-HSD) [56,57] and hydroxysteroid sulfotransferase (HST) [58] [25,26, for reviews] (Fig. 1), has proven to be a very suitable model for studying the regulation of the production of neuroactive steroids [49,59–64]. In the present work, we have thus used frog hypothalamic explants and homogenates to investigate the effect and mechanism of action of etifoxine on neurosteroid biosynthesis.

## Materials and Methods

### Animals

Adult male frogs (*Rana esculenta*; body weight ranging from 30 to 40 g) were obtained from a commercial source (Couétard, Saint-Hilaire de Riez, France). The animals were maintained under a 12-h light, 12-h dark schedule (lights on from 06:00–18:00 h) in a temperature-controlled room (8 ± 0.5°C). Frogs were kept under running water for at least one week before being sacrificed. In order to limit possible variations of neurosteroid biosynthesis due to circadian rhythms [65], all animals were killed between 09:30 and 10:30 a.m. Frogs were anesthetized in 0.1% 3-amino-benzoic acid ethyl ester (MS222) solution and sacrificed by decapitation. This study was carried out in strict accordance with the recommendations of the Directive 2010/63/EU of the European Parliament and of the Council of September 22, 2010 on the protection of animals used for scientific purposes, published in the Official Journal of the European Union L276/33 (20.10.2010). The protocol was approved by the French Local Ethical Committee of Normandy (CENOMEXA; approval number N/01-09-07/07/09-10) and conducted under the supervision of authorized investigators (JL do Rego; authorization no. 76/08/015 from the Ministère de l'Ecologie et du Développement Durable).

### Chemicals and reagents

Tritiated Δ^5^P ([^3^H]Δ^5^P) (specific activity 14 Ci/mmol), tritiated DHEA ([^3^H]DHEA), tritiated androstenedione ([^3^H]Δ^4^), tritiated P ([^3^H]P), tritiated THP ([^3^H]THP), tritiated tetrahydrodeoxycorticosterone ([^3^H]THDOC) and tritiated 17-hydroxyprogesterone ([^3^H]17OH-P) were purchased from Perkin Elmer (Paris, France). DHP was purchased from steraloids (Wilton, NH, USA). 17-hydroxypregnenolone (17OH-Δ^5^P), bicuculline, DL-aminoglutethimide, flumazenil (Ro15-1788), N-2-hydroxy-ethyl-piperazine-N’-2-ethane sulfonic acid (HEPES), PK11195, propylene glycol, trifluoroacetic acid (TFA) were from Sigma-Aldrich (St. Louis, MO). Triakontatetraneuropeptide (TTN) was obtained from PolyPeptide Laboratories (Strasbourg, France). Etifoxine hydrochloride (batches 403, 439 and 508) was a gift from Biocodex (Compiègne, France). Methanol and dichloromethane were from Carlo Erba (Val-de-Reuil, France). Bovine serum albumin (BSA) was from Boerhinger (Paris, France).

### Measurement of steroidogenic enzyme activities in brain tissue explants

The experimental procedure applied to study the conversion of [^3^H]Δ^5^P into different metabolites has been previously described [55,61,62]. Briefly, for each experimental value, the hypothalami from 4 frogs (approximately 10 mg of tissue) were rapidly dissected out and each hypothalamus was cut into 2 halves. The tissue fragments were preincubated for 15 min in 1 ml of Ringer’s solution consisting of 15 mM HEPES buffer, 112 mM NaCl, 15 mM NaHCO_3_, 2 mM CaCl_2_, 2 mM KCl, supplemented with 2 mg glucose/ml and 0.3 mg BSA/ml. The incubation medium was gassed with a 95% O_2_/5% CO_2_ mixture and the pH was adjusted to 7.4. The hypothalamic explants were incubated at 24°C for 2 h (0.25 to 4 h for time-course experiments) in 500 μl Ringer’s medium containing 10^-6^ M [^3^H]Δ^5^P and 4% propylene glycol, in the absence or presence of test substances. In order to avoid a possible interference of endogenous Δ^5^P in the conversion of [^3^H]Δ^5^P into tritiated neurosteroids, aminoglutethimide (10^-5^ M), a specific inhibitor of the cholesterol side-chain cleavage enzyme P450scc, was added to the incubation medium. Aminoglutethimide, which is poorly soluble in water, was dissolved in methanol (0.1%), and the same concentration of CH_3_-OH was added in control samples. At the end of the incubation period, the tissues were rinsed 4 times with ice-cold Ringer’s buffer and the reaction was stopped by adding 1 ml of trichloroacetic acid. The tissues were homogenized with a glass potter homogenizer, and the steroids were extracted three times by 1 ml of dichloromethane. The organic phase containing the steroids was evaporated under nitrogen and the tissue extracts were dissolved in a solution consisting of 65% water/TFA (99.9:0.1; vol/vol; sol. A) and 35% methanol/water/TFA (90:9.98:0.02; vol/vol/vol; sol. B) and pre-purified on Sep-Pak C_18_ cartridges (Waters Associates, Milford, MA) equilibred with a solution made of 65% sol. A and 35% sol. B. Steroids were eluted with 4 ml of a solution made of 10% sol. A and 90% sol. B. The solvent was evaporated in a Speed-Vac concentrator (Savant, Hicksville, NY) and the extracts were kept dry at 4°C until RP-HPLC analysis.

### Measurement of steroidogenic enzyme activities in brain tissue homogenates

For each experimental value the hypothalami from 4 frogs were rinsed in 1 ml of Ringer’s medium previously gassed with a 95% O_2_/5% CO_2_ mixture and the pH was adjusted to 7.4. The tissues were homogenized with a glass Potter homogenizer in 480 μl Ringer’s medium containing 10^-5^ M aminoglutethimide and the homogenate was incubated at 24°C for 15 min to 4 h with 10^-6^ M [^3^H]Δ^5^P supplemented with 4% propylene glycol, in the absence or presence of test substances. At the end of the incubation period, the reaction was stopped by adding 500 μl of ice-cold trichloroacetic acid and transferring the tubes into a cold water bath (0°C). Steroids were extracted three times with 1 ml of dichloromethane and pre-purified on Sep-Pak C_18_ cartridges as described above.

### High performance liquid chromatography

Sep-Pak-prepurified brain tissue and homogenate extracts were analyzed by RP-HPLC as previously described [55,61,62] using a Gilson model 305 master pump acting as a system controller, a Gilson model 306 slave pump controlled by the previous pump, a Gilson model 115 variable wavelenght UV detector set at 240 nm (Gilson S.A., Villier-le-Bel, France) and a Rheodyne model 7125 injector (Rheodyne Inc, California). A 0.39 X 30 cm Nova-Pak C_18_ column (Waters Associates) equilibrated with 60% sol. A and 40% sol. B was used for analysis. Each dry extract was dissolved in 400 μl of a solution consisting of 60% sol. A and 40% sol. B, and the whole sample was injected at a flow rate of 1 ml/min. The radioactive steroids formed from [^3^H]Δ^5^P were separated using a gradient of sol. B (40–100% over 104 min) including 4 isocratic steps at 40% (0–10 min), 64% (39–59 min), 80% (69–79 min) and 100% sol. B (94–104 min). Tritiated compounds eluted from the HPLC column were detected by using a flow scintillation analyzer (Radiomatic Flo-One\Beta A-500, Packard, Meridien, CT) and the radioactivity contained in each peak was integrated.

Synthetic steroids used as reference standards were chromatographed under the same conditions as the tissue and homogenate extracts, and their elution positions were determined by liquid scintillation (tritiated standards) or by UV absorption (non radioactive standards).

### Quantification of steroid biosynthesis and statistical analysis

The amounts of radioactive steroids formed by conversion of [^3^H]Δ^5^P were expressed as a percentage of the total radioactivity contained in all peaks resolved by RP-HPLC including [^3^H]Δ^5^P itself. Each value is the mean of 4 independent experiments from distinct hypothalamic extracts. Statistical analysis was performed by ANOVA followed by Dunnett’s or Student-Newman-Keul’s multiple comparison test.

## Results

### Effect of etifoxine on neurosteroid biosynthesis by brain tissue explants

**Fig 2:**
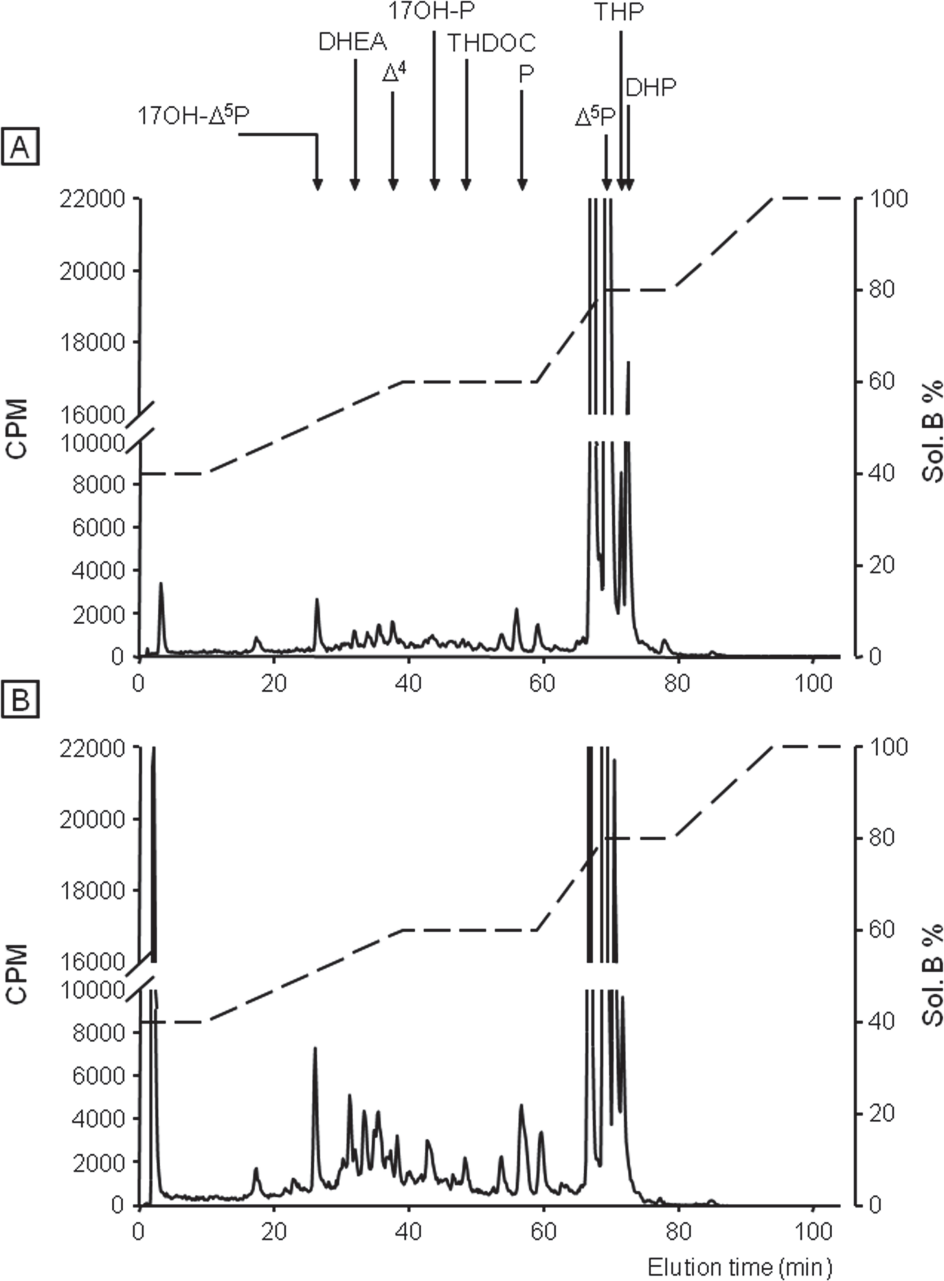
Analysis of radioactive steroids formed after a 2-h incubation of frog hypothalamic explants with tritiated pregnenolone ([^3^H]Δ^5^P) in the absence (A) or presence of 3x10^-6^ M etifoxine (B). The ordinate indicates the radioactivity measured in the HPLC eluent. The dashed lines represent the gradient of secondary solvent (% solution B). The arrows indicate the elution positions of standard steroids: 17OH-**Δ** ^5^P, 17-hydroxypregnenolone; DHEA, dehydroepiandrosterone; **Δ** ^4^, androstenedione; 17OH-P, 17-hydroxyprogesterone; tetrahydrodeoxycorticosterone, THDOC; P, progesterone; **Δ** ^5^P, pregnenolone; DHP, dihydroprogesterone; THP, tetrahydroprogesterone.

**Fig 3:**
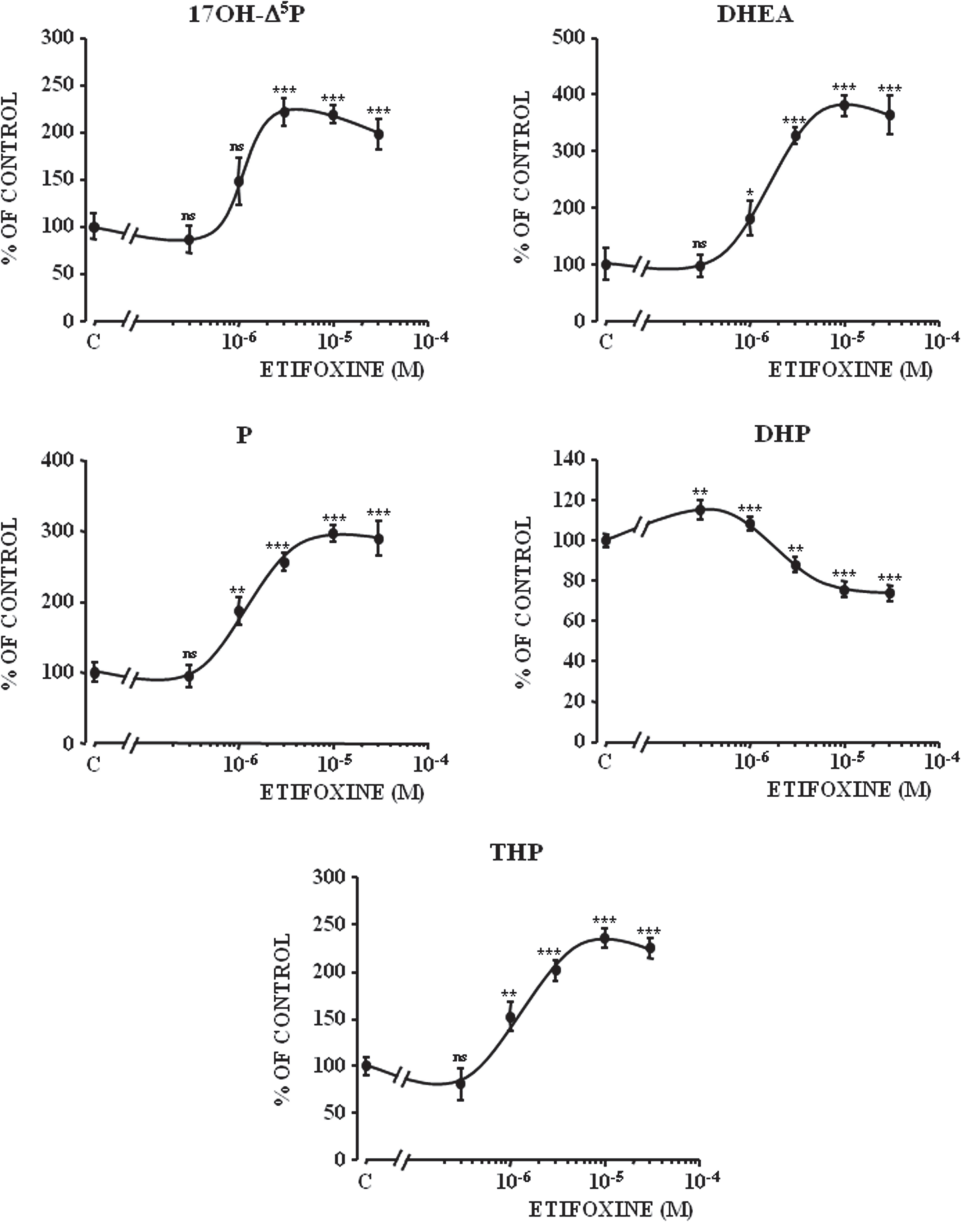
Effect of graded concentrations of etifoxine on the conversion of tritiated pregnenolone ([^3^H]Δ^5^P) into 17-hydroxypregnenolone (17OH-Δ^5^P), dehydroepiandrosterone (DHEA), progesterone (P), dihydroprogesterone (DHP) and tetrahydroprogesterone (THP) by frog hypothalamic explants (duration of the incubation: 2h). The values were calculated from the areas under the peaks in chromatograms similar to those presented in Fig. 1. Results are expressed as percentages of the amount of each steroid formed in the absence of etifoxine. Values are the mean (± SEM) of four independent experiments. *p<0.05; **p<0.01; ***p<0.001; ns, not statistically different from control (C).

Incubation of frog hypothalamic explants with [^3^H]**Δ** ^5^P used as a steroid precursor, combined with reversed phase HPLC analysis coupled to flow scintillation detection, was performed to study the possible effect of etifoxine on the biosynthesis of neurosteroids. After a 2-h exposure period of hypothalamic explants with [^3^H]**Δ** ^5^P, the HPLC gradient used made it possible to resolve several radioactive metabolites which exhibited the same retention time as 17OH-**Δ** ^5^P, DHEA, androstenedione (**Δ** ^4^), 17OH-P, THDOC, P, THP and DHP (Fig. 2A). Addition of etifoxine at a concentration of 3x10^-6^ M to the incubation medium markedly stimulated the conversion of [^3^H]**Δ** ^5^P into radioactive 17OH-**Δ** ^5^P, DHEA, **Δ** ^4^, 17OH-P, THDOC, P and THP (Fig. 2B). In contrast, etifoxine provoked a decrease of the formation of DHP, an intermediate product in the biosynthetic pathway of THP (Fig. 2B). Incubation of hypothalamic explants with graded concentrations of etifoxine (3x10^-7^ to 3x10^-5^ M) induced a dose-dependent increase in the production of 17OH-**Δ** ^5^P, DHEA, P and THP (Fig. 3). At the highest concentrations tested (3x10^-6^ to 3x10^-5^ M), etifoxine concomitantly inhibited the biosynthesis of DHP (Fig. 3). The maximum effects were observed at a dose 10^-5^ M.

**Fig 4:**
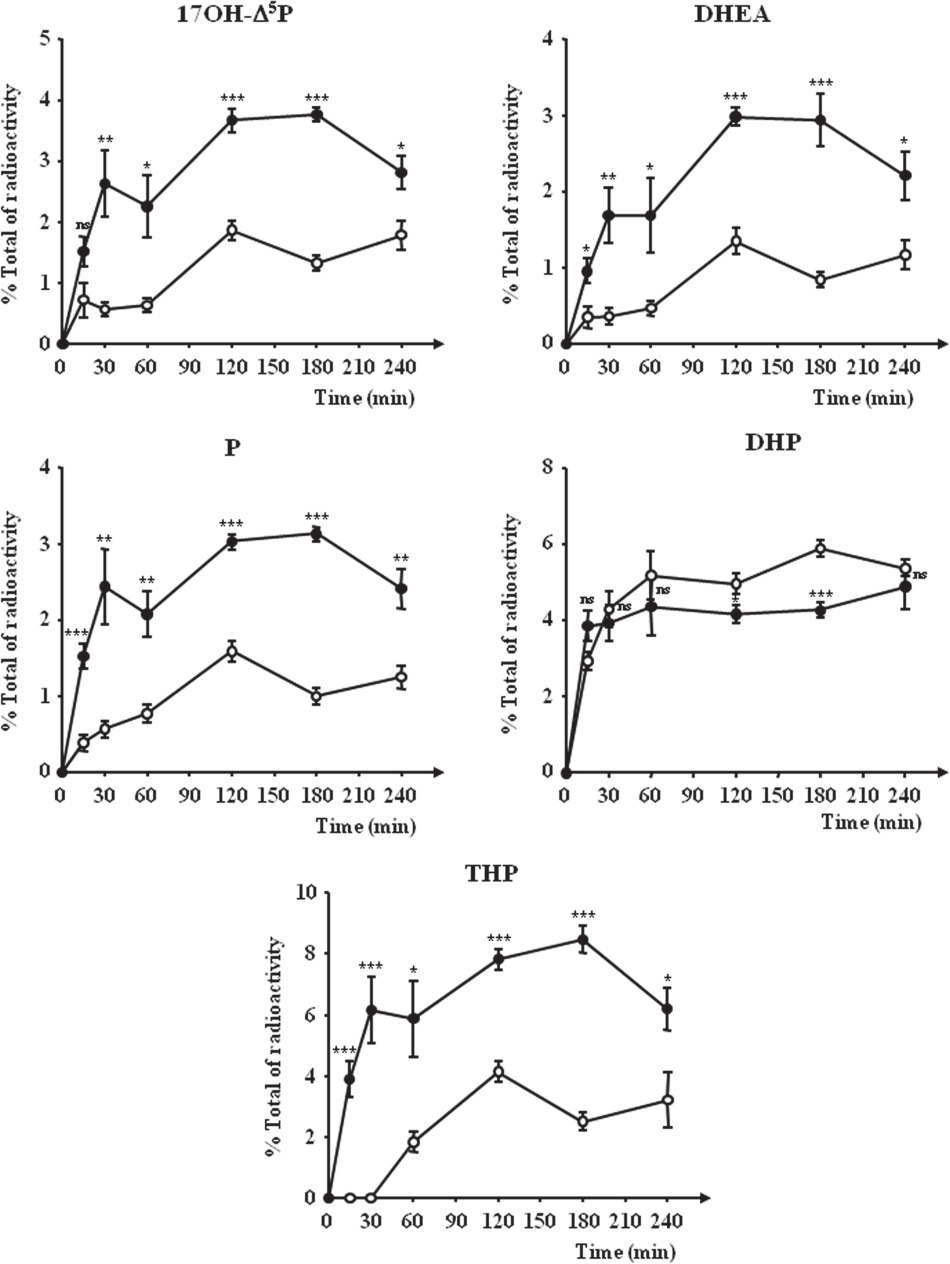
Time-course of the conversion of tritiated pregnenolone ([^3^H]Δ^5^P) into radioactive 17-hydroxypregnenolone (17OH-Δ^5^P), dehydroepiandrosterone (DHEA), progesterone (P), dihydroprogesterone (DHP) and tetrahydroprogesterone (THP) by frog hypothalamic explants in the absence (○) or presence of 3x10^-6^ M etifoxine (●). The values were calculated from the areas under the peaks in chromatograms similar to those presented in Fig. 1. Results are expressed as percentages of the amount of each steroid formed compared to the total amount of radiolabeled compounds resolved by HPLC analysis including [^3^H]**Δ** ^5^P. Values are the mean (± SEM) of four independent experiments. *p<0.05; **p<0.01; ***p<0.001 compared to respective control values; ns, not statistically different (one-way ANOVA followed by a *post hoc* Dunnett’s test).

Time-course investigations revealed that a 15-min incubation of frog hypothalamic explants with etifoxine (3x10^-6^ M) was sufficient to induce a significant stimulation of the biosynthesis of DHEA, P and THP (Fig. 4). The maximum response was observed after 2–3 h of exposure; then, the stimulatory effect of etifoxine slightly declined during the next hour (Fig. 4). In contrast, the inhibitory effect of etifoxine on DHP biosynthesis was only detectable 2 h after the onset of the incubation period (Fig. 4).

**Fig 5:**
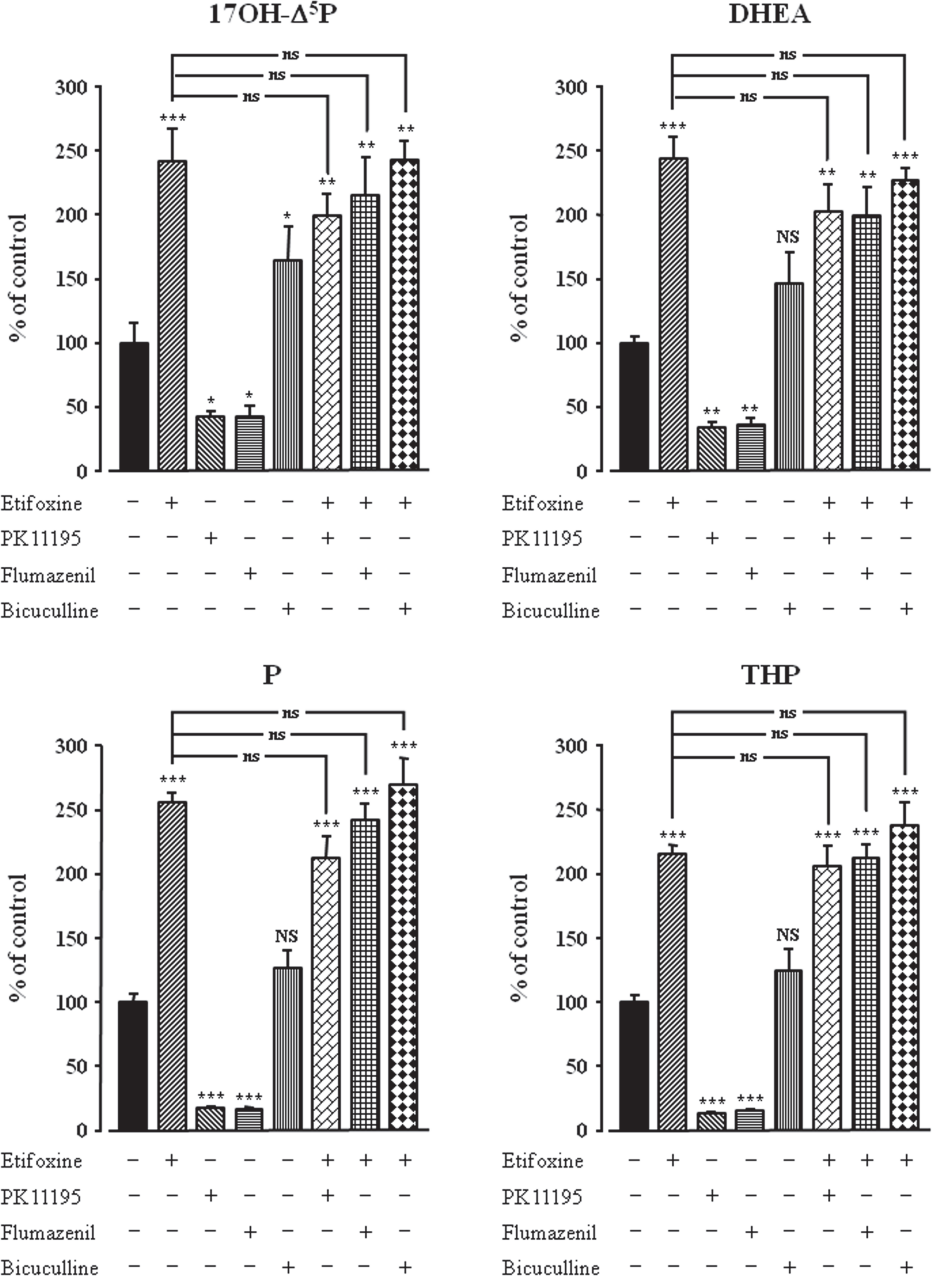
Effects of etifoxine (3x10^-6^ M) in the absence or presence of the TSPO antagonist PK11195 (3x10^-5^ M), the central-type benzodiazepine receptor antagonist flumazenil (3x10^-5^ M) or the GABA_A_ receptor antagonist bicuculline (3x10^-5^ M) on the conversion of tritiated pregnenolone ([^3^H]Δ^5^P) into 17-hydroxypregnenolone (17OH-Δ^5^P), dehydroepiandrosterone (DHEA), progesterone (P) and tetrahydroprogesterone (THP) by frog hypothalamic explants. The values were obtained from experiments similar to those presented in Fig. 1. Results are expressed as percentages of the amount of each steroid formed in the absence of drugs. Values are the mean (± SEM) of four independent experiments. *p<0.05; **p<0.01; ***p<0.001 compared to respective control values; NS, not statistically different from control; ns, not statistically different from etifoxine-stimulated level (one-way ANOVA followed by a *post hoc* Student-Newman-Keul’s test).

**Fig 6:**
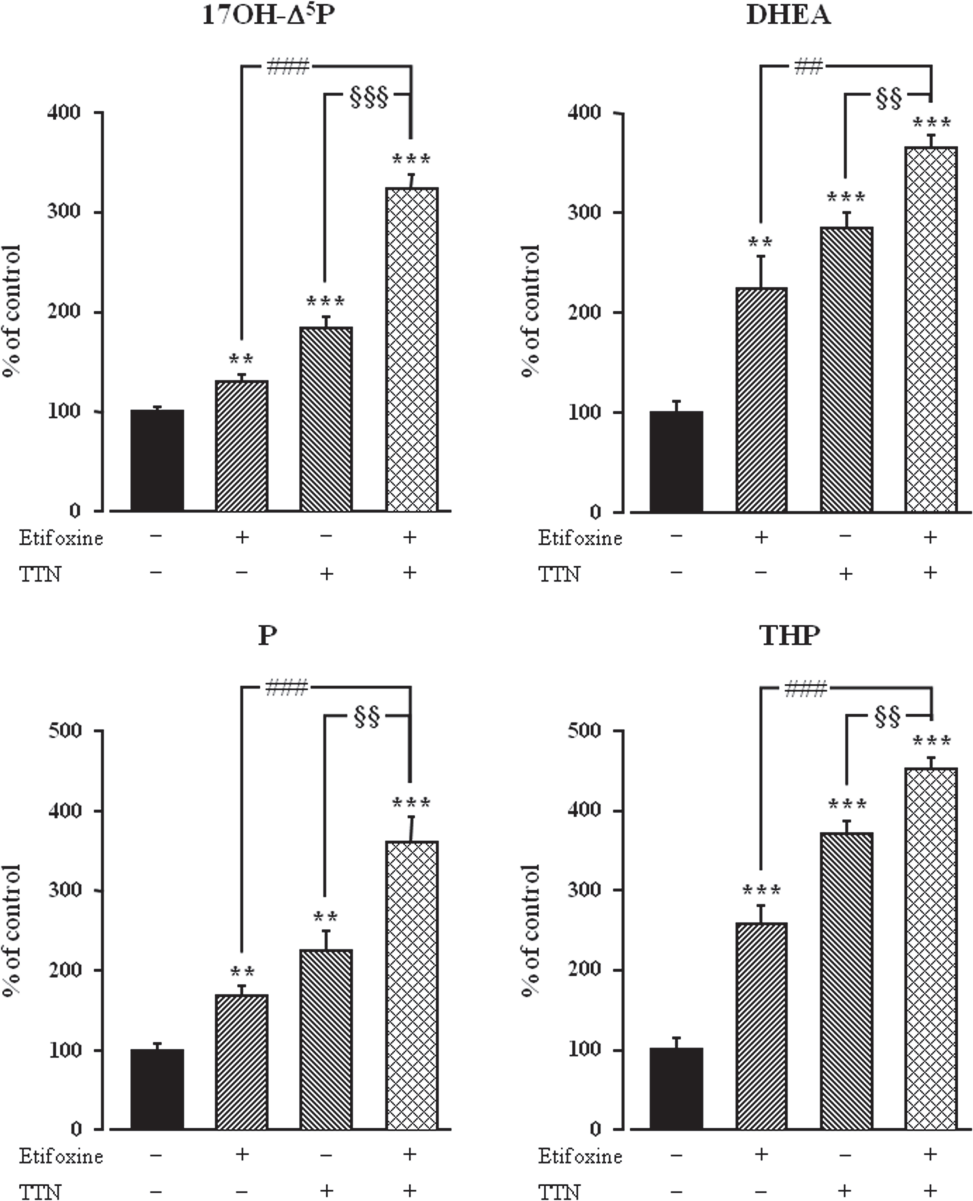
Effects of etifoxine (10^-6^ M) in the absence or presence of triakontatetraneuropeptide (TTN), a specific TSPO agonist (3x10^-8^ M), on the conversion of tritiated pregnenolone ([^3^H]Δ^5^P) into 17-hydroxypregnenolone (17OH-Δ^5^P), dehydroepiandrosterone (DHEA), progesterone (P) and tetrahydroprogesterone (THP) by frog hypothalamic explants. The values were obtained from experiments similar to those presented in Fig. 1. Results are expressed as percentages of the amount of each steroid formed in the absence of drugs. Each value is the mean (± SEM) of four independent experiments. **p<0.01, ***p<0.001 vs control; ##*p*<0.01, ###*p*<0.001 vs etifoxine alone; ^§§^ *p*<0.01, ^§§§^ *p*<0.001 vs TTN alone (one-way ANOVA followed by a *post hoc* Student-Newman-Keul’s test).

The mode of action of etifoxine on neurosteroid production by frog hypothalamic explants was investigated using selective TSPO and CBR antagonists. As previously reported [49,60], the TSPO antagonist PK11195 (3x10^-5^ M) and the CBR antagonist flumazenil (3x10^-5^ M), both induced by themselves a significant inhibition of the conversion of [^3^H]**Δ** ^5^P into 17OH-**Δ** ^5^P, DHEA, P and THP (Fig. 5). However, neither PK11195 nor flumazenil significantly affected the stimulatory action of etifoxine (3x10^-6^ M) on neurosteroid biosynthesis (Fig. 5). Consistent with previous data [59], bicuculline alone (3x10^-5^ M) provoked a modest stimulation of neurosteroid formation. Nevertheless, bicuculline did not significantly modify the neurosteroidogenic response to etifoxine (Fig. 5). As previously shown [49], the specific TSPO agonist TTN (3x10^-8^ M) provoked a robust increase in the biosynthesis of neurosteroids by hypothalamic explants. Co-administration of TTN and etifoxine revealed that the stimulatory effects of both compounds on the conversion of [^3^H]**Δ** ^5^P into radioactive 17OH-**Δ** ^5^P, DHEA, P and THP by hypothalamic explants were significantly higher than the effects exerted individually by etifoxine or TTN (Fig. 6).

### Effect of etifoxine on neurosteroid biosynthesis by brain tissue homogenates

**Fig 7:**
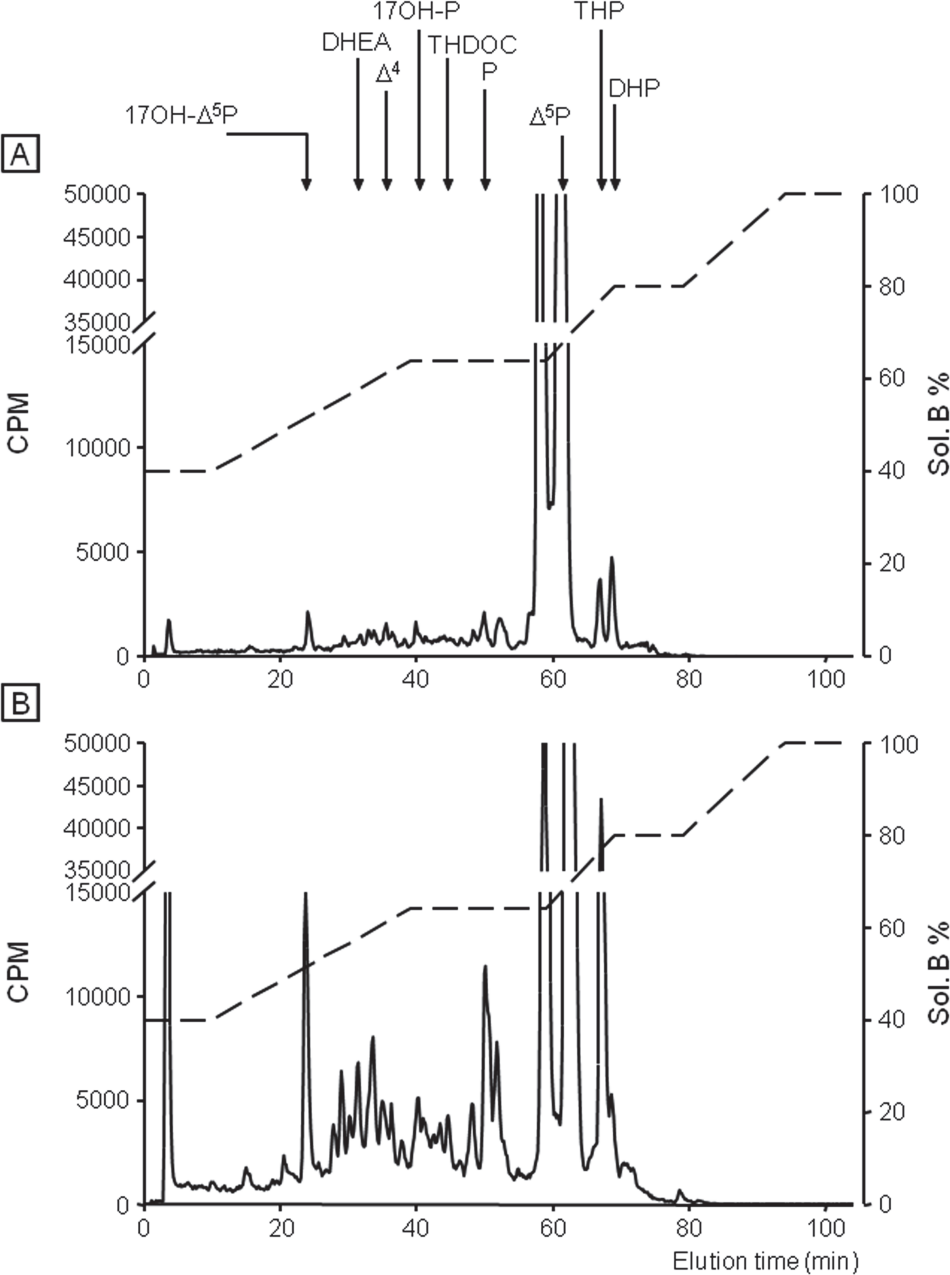
HPLC analysis of radioactive steroids formed after a 1-h incubation of frog hypothalamic homogenates with tritiated pregnenolone ([^3^H]Δ^5^P) in the absence (A) or presence of 10^-6^ M etifoxine (B). The ordinate indicates the radioactivity measured in the HPLC eluent. The dashed lines represent the gradient of secondary solvent (% solution B). The arrows indicate the elution positions of standard steroids: 17OH-**Δ** ^5^P, 17-hydroxypregnenolone; DHEA, dehydroepiandrosterone; **Δ** ^4^, androstenedione; 17OH-P, 17-hydroxyprogesterone; tetrahydrodeoxycorticosterone, THDOC; P, progesterone; **Δ** ^5^P, pregnenolone; DHP, dihydroprogesterone; THP, tetrahydroprogesterone.

**Fig 8:**
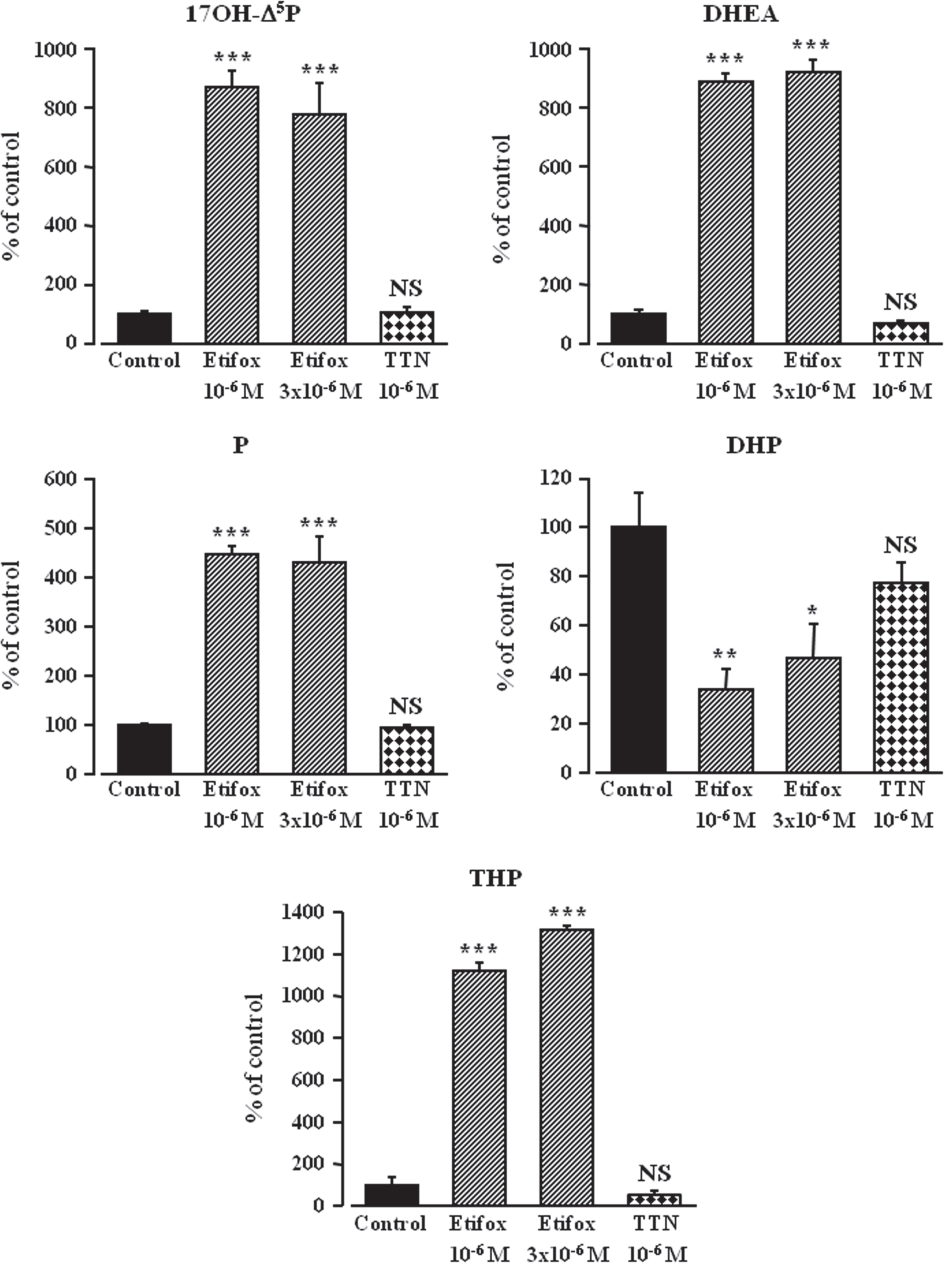
Effects of etifoxine (10^-6^ M and 3x10^-6^ M) or triakontatetraneuropeptide (TTN, 10^-6^ M) on the conversion of tritiated pregnenolone into 17-hydroxypregnenolone (17OH-Δ^5^P), dehydroepiandrosterone (DHEA), progesterone (P), dihydroprogesterone (DHP) and tetrahydroprogesterone (THP) by frog hypothalamic homogenates (duration of the incubation: 1h). The values were calculated from the areas under the peaks in chromatograms similar to those presented in Fig. 6. Results are expressed as percentages of the amount of each steroid formed in the absence of etifoxine. Values are the mean (± SEM) of four independent experiments. *p<0.05; **p<0.01; ***p<0.001; NS, not statistically different from control (C).

To look for a possible direct effect of etifoxine on neurosteroid biosynthesis, we next used tissue homogenates, a preparation in which membrane receptor signaling is disrupted. A 1-h incubation of frog hypothalamic homogenates with [^3^H]**Δ** ^5^P yielded to the formation of various radioactive steroids (Fig. 7A). In the presence of etifoxine (10^-6^ M), the production of neurosteroids by hypothalamic homogenates was strongly enhanced (Fig. 7B). At the two concentrations tested (10^-6^ and 3x10^-6^ M) etifoxine provoked a significant increase in the neosynthesis of tritiated 17OH-**Δ** ^5^P, DHEA, P and THP associated with a decrease in the formation of DHP (Fig. 8). In contrast to what was observed with hypothalamic explants (Fig. 6), TTN did not affect neurosteroid biosynthesis in hypothalamic homogenates (Fig. 8).

**Fig 9:**
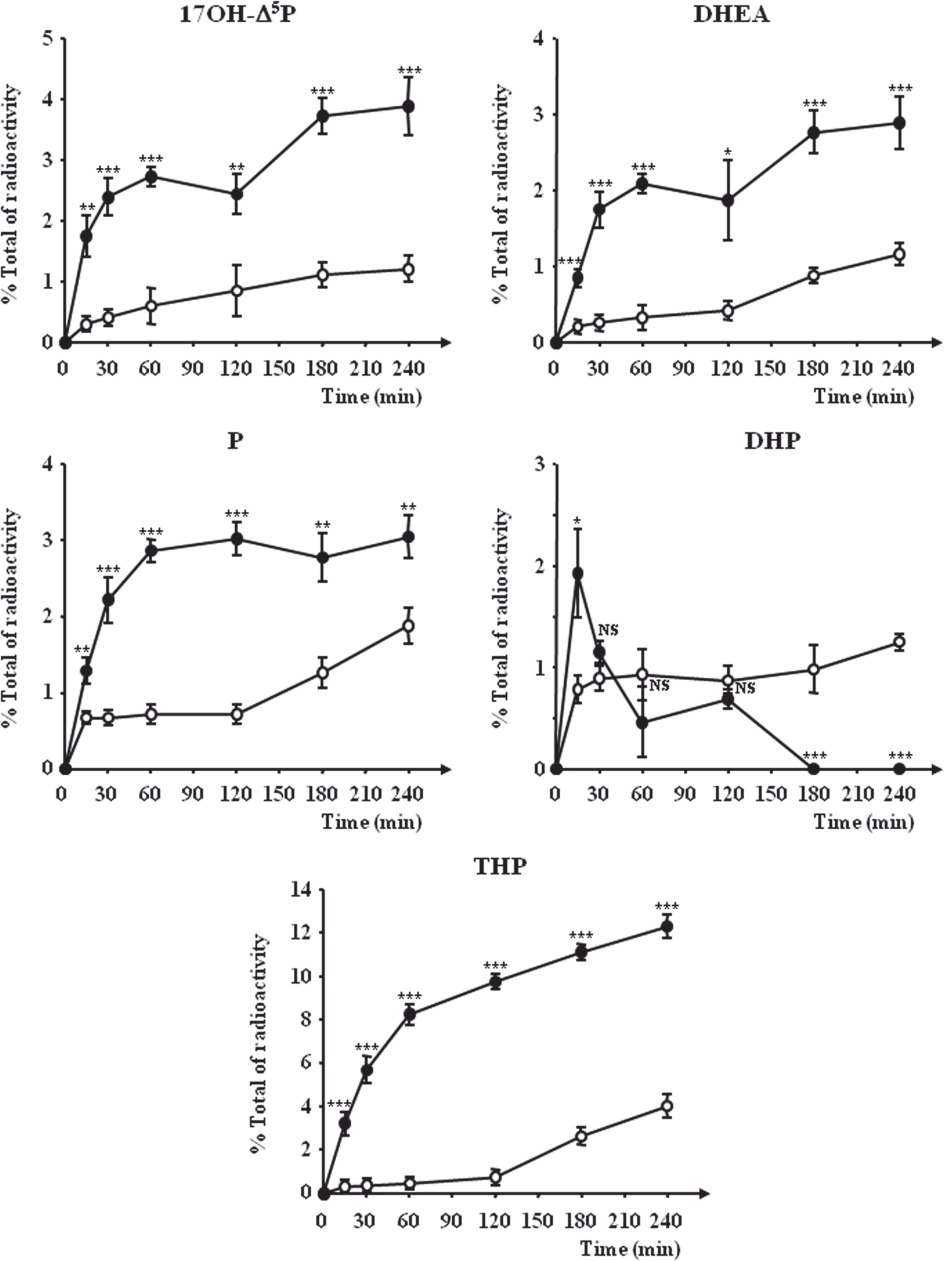
Time-course of the conversion of tritiated pregnenolone ([^3^H]Δ^5^P) into radioactive 17-hydroxypregnenolone (17OH-Δ^5^P), dehydroepiandrosterone (DHEA), progesterone (P), dihydroprogesterone (DHP) and tetrahydroprogesterone (THP) by frog hypothalamic homogenates in the absence (○) or presence of 10^-6^ M etifoxine (●). The values were calculated from the areas under the peaks in chromatograms similar to those presented in Fig. 6. Results are expressed as percentages of the amount of each steroid formed compared to the total amount of radiolabeled compounds resolved by HPLC analysis including [^3^H]**Δ** ^5^P. Values are the mean (± SEM) of four independent experiments. *p<0.05; **p<0.01; ***p<0.001 compared to respective control values; NS, not statistically different (one-way ANOVA followed by a post hoc Dunnett’s test).

Kinetic experiments showed that etifoxine (10^-6^ M) induced within 15 min a significant increase of the biosynthesis of 17OH-**Δ** ^5^P, DHEA, P and THP by hypothalamic homogenates (Fig. 9). While etifoxine provoked a sustained stimulation of 17OH-**Δ** ^5^P, DHEA, P and THP, the effect on DHP was transient and gradually declined (Fig. 9). After 3h exposure to etifoxine, [^3^H]DHP was no longer present in the incubation medium (Fig. 9).

## Discussion

Behavioral and neurophysiological studies have revealed that the anxiolytic and neurotrophic activities of etifoxine may be mediated, at least in part, through increased production of neuroactive steroids [13,14,18,38]. However, the molecular mechanism by which etifoxine can stimulate neurosteroid biosynthesis remains poorly understood. In this context, uncovering the mode of action of etifoxine on nerve cells expressing steroidogenic enzymes requires a sensitive and specific approach. By combining incubation of frog hypothalamic explants or homogenates in the presence of a radioactive steroid precursor with HPLC analysis and continuous flow scintillation [59–61], we here demonstrate that etifoxine triggers the activity of various steroidogenic enzymes through a membrane receptor-independent mechanism.

We first showed that etifoxine induces a concentration-dependent increase in the formation of several steroids, including 17OH-Δ^5^P, DHEA, P, and THP, and a concomitant decrease in the production of DHP that can probably be accounted for by the conversion of the latter into THP. In steroidogenic cells, DHP is synthesized from P through the action of 5α-R, whereas the formation of THP is catalyzed by 3α-HSD, a bifunctional enzyme that interconverts, in a reversible manner, DHP into THP (Fig. 1). The increase of THP induced by etifoxine can thus be ascribed to either stimulation of the reduction reaction of DHP into THP, or inhibition of the oxidation reaction of THP into DHP. Our data indicate that etifoxine stimulates the biological activity of certain steroidogenic enzymes, such as 3β-HSD, P450_C17_, 5α-R and/or 3α-HSD in frog hypothalamic neurons. Consistent with this observation, *in vivo* studies have previously shown that intra-peritoneal administration of etifoxine causes an increase in the brain content of **Δ** ^5^P, P and THP in adrenalectomized and castrated rats [18]. In addition, it has been reported that neurosteroidogenic enzyme inhibitors such as trilostane, a specific inhibitor of 3β-HSD [66], finasteride, an inhibitor of 5α-R [67] and indomethacin, an inhibitor of 3α-HSD [68] suppress the anxiolytic effect of etifoxine [38].

Interestingly, kinetic experiments showed that a 15-min exposure of hypothalamic explants to etifoxine was sufficient to induce a robust increase in neurosteroid synthesis. This rapid change implies that etifoxine does not activate steroidogenic enzyme gene transcription but rather acts at a post-translational level, likely through serine (Ser) and/or threonine (Thr) phosphorylation of the enzymes. In particular, it is clearly established that phosphorylation of of Ser^106^ and Thr^112^ residues in human P450_C17_ stimulates the activity of the enzyme [69–74]. Interestingly, a rapid response in the activity of 3α-HSD has been observed in the rat brain after administration of fluoxetine [75–78], which like etifoxine exerts anxiolytic properties [79,80].

The anxiolytic effects of etifoxine have been ascribed either to its potentiating action on GABAergic transmission at the GABA_A_ receptor level [3,81] or to an indirect interaction involving the activation of TSPO [3,18] while the neurotrophic effects of etifoxine appear to be mediated through TSPO via the production of neurosteroids [13,14]. Since CBR and TSPO agonists stimulate neurosteroid production in the frog hypothalamus [49,60], we have hypothesized that the action of etifoxine on neurosteroidogenesis could be mediated through either the GABA_A_/CBR complex or TSPO. However, the specific CBR antagonist flumazenil and the specific TSPO antagonist PK11195, which both reduced basal neurosteroid biosynthesis, did not abolish the stimulatory effect of etifoxine on the conversion of [^3^H]Δ^5^P into radioactive neurosteroids. Similarly, the selective GABA_A_ receptor antagonist bicuculline did not modify etifoxine-induced neurosteroid production. These data indicate that the action of etifoxine on neurosteroid synthesis is not mediated through activation of GABA_A_/CBR or TSPO. In support of this notion, we found that etifoxine and TTN (a TSPO agonist) exert additive effects on neurosteroidogenesis indicating that these two molecules act via distinct mechanisms. Overall, these observations indicated that etifoxine could exert its effects on neurosteroid-producing cells either through a receptor different from GABA_A_/CBR and TSPO, or through a direct action on the activity of steroidogenic enzymes in the central nervous systems. In any event, the fact that CBR and TSPO antagonits *per se* caused a marked inhibition of neurosteroid biosynthesis but did not modify the stimulatory effect of etifoxine, suggests that this compound exerts its action dowstream of CBR and TSPO.

To determine whether the etifoxine-induced stimulation of neurosteroid production depends on activation of a membrane receptor, we next used hypothalamic tissue homogenates, a preparation in which plasma membrane receptor signaling is disrupted. We found that a 1-h incubation of hypothalamic homogenates with etifoxine strongly activated the conversion of [^3^H]Δ^5^P into radioactive 17OH-Δ^5^P, DHEA, P, and THP, whereas the synthesis of DHP significantly decreased. Of note, the increase in neurosteroid biosynthesis induced by etifoxine was 3–4 times higher in hypothalamic homogenates than in hypothalamic explants and the maximum response was observed at a concentration of 10^-6^ M etifoxine in hypothalamic homogenates vs 10^-5^ M in hypothalamic explants. In contrast, TTN, which exerts its stimulatory action on the formation of neurosteroids through activation of TSPO [49], did not affect neurosteroidogenesis in hypothalamic homogenates. The tissue homogenates probably contained intact mitochondria harboring TSPO which mediates the translocation of cholesterol from the outer mitochondrial membrane to the inner mitochondrial membrane [82–85], where it is converted into Δ^5^P by P450scc [86,87] (Fig. 1). Once formed, Δ^5^P diffuses from mitochondria to the cytoplasm where it is converted to P by 3β-HSD, and to 17OH-Δ^5^P by P450_C17_. However, in the present study, tritiated Δ^5^P was used as a precursor, and the synthesis of endogenous Δ^5^P was blocked by aminoglutethimide, a specific inhibitor of the enzyme P450scc. Thus, the occurrence of intact mitochondria possessing active TSPO in hypothalamic homogenates could not have any influence on the conversion of Δ^5^P into neuroactive steroids. Taken together, these data clearly indicate that the stimulatory effect of etifoxine on neurosteroid biosynthesis is not mediated via a membrane receptor. Time-course experiments conducted with brain homogenates, revealed that etifoxine induced a significant increase in neurosteroid biosynthesis within 15 min, confirming that the compound activates steroidogenic enzymes at a post-translational level.

Behavioral and neurochemical studies indicate that THP and DHEA exert anxiolytic and antidepressant effects [21,27–36] while Δ^5^P and P facilitate nerve regeneration [39–43]. The fact that etifoxine directly stimulates the formation of THP, DHEA and P thus strongly suggests that the anxiolytic and neuroprotective effects of etifoxine can be ascribed to its ability to activate the biosynthesis of neurosteroids. Nevertheless, we cannot exclude that the binding of etifoxine to and the subsequent activation of TSPO contribute also in part to enhancement of neurosteroid biosynthesis as shown in other experimental models [3,18].

In conclusion, the present study provides the first direct evidence that etifoxine stimulates neurosteroid biosynthesis in the central nervous system of vertebrates. These findings support the view that the anxiolytic and neuroprotective actions of etifoxine are mediated, at least in part, through enhanced production of neurosteroids. Our data also indicate that the action of etifoxine does not implicate a membrane receptor but can be accounted for by direct stimulation of steroidogenic enzyme activity at a post-translational level.
